# Cascade mentoring: a proposal for transforming radiology education in line with the European training curriculum

**DOI:** 10.1186/s13244-026-02317-1

**Published:** 2026-06-06

**Authors:** Alberto Vieira, Ana Catarina Vieira

**Affiliations:** 1 FMUP–Faculdade de Medicina, Universidade do Porto, Porto, Portugal; 2 Hospital CUF Porto, Porto, Portugal

**Keywords:** Radiology, Education (medical, graduate), Mentoring, Teaching, Curriculum

## Abstract

**Abstract:**

Although mentoring remains a fundamental part of medical training, the traditional approach of a senior mentor guiding a junior trainee often fails to meet the diverse and evolving needs of learners in radiology. While peer and near-peer mentoring approaches have shown promise, they often lack a scalable, structured framework. We present a cascade mentoring model embedded within the radiology training environment, in which senior trainees mentor junior residents, who then supervise undergraduate learners. This model is aligned with the European Society of Radiology’s (ESR) competency-based framework through the European training curriculum for radiology (ETC). It sets out the specific responsibilities of faculty members, early career radiologists, senior residents, junior residents and undergraduates. Mentorship activities include case discussions, clinical teaching, research collaboration and providing structured feedback. The cascade approach fosters skill acquisition, professional development and research engagement at every level. Senior residents can refine their expertise through teaching, junior residents can reinforce their foundational knowledge by guiding undergraduates, and undergraduates can gain structured, early exposure to radiology. Anticipated benefits include enhanced departmental integration, improved teaching skills among residents, greater undergraduate engagement and an optimised faculty workload. The Cascade Mentoring Model is an innovative, sustainable educational strategy that is aligned with the ETC. It empowers residents to become future educators and promotes radiology as a speciality. When implemented, overseen and evaluated deliberately, it has the potential to enhance radiology education programmes throughout Europe. Cascade mentoring can also help mitigate the shortage of radiologists by enhancing training capacity and retention of early-career radiologists.

**Critical relevance statement:**

The cascade mentoring model provides a structured, scalable approach to radiology education aligned with the European training curriculum. By optimising faculty workload, strengthening residents’ teaching skills and fostering early engagement, it supports high-quality training, workforce retention and sustainable radiology education across Europe.

**Key Points:**

- Traditional mentoring in radiology lacks scalability and responsiveness to learner diversity needs.
- Cascade mentoring structures near-peer teaching across undergraduate, resident and faculty levels.
- Model aligns with the ESR European training curriculum competency-based framework.
- Cascade mentoring enhances skills, teaching capacity, research engagement and professional development.
- Scalable approach supports workforce retention and sustainable radiology education in Europe.

**Graphical Abstract:**

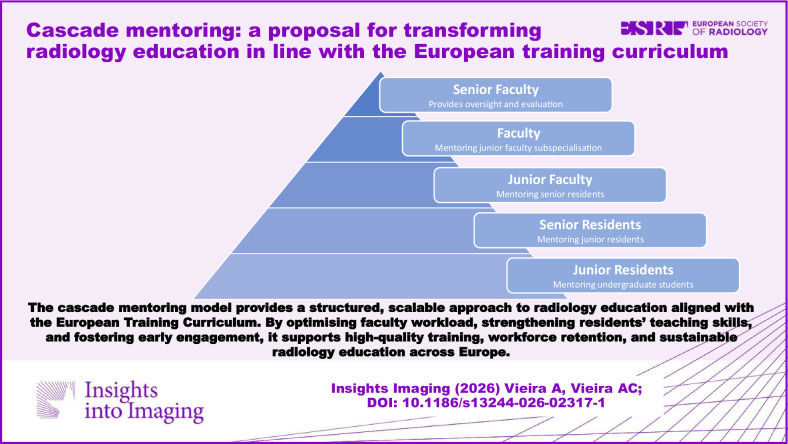

## Introduction

Radiology has become a crucial medical specialty, driven by the growing need for precise and reliable diagnosis of medical conditions.

Radiology education in Europe is guided by the structured European training curriculum for Radiology (Levels I–III), which was developed by the European Society of Radiology [1, 2].

This curriculum outlines the fundamental knowledge, skills and professionalism required, as well as the training duration, structure and levels of subspecialisation. However, the rapid advancement of imaging technologies, the growing trend towards subspecialisation and the shortage of qualified faculty are placing increasing strain on existing training sites across Europe [3]. For instance, the clinical radiology workforce in the UK is growing at a rate of around 4–6% per year, but not sufficiently to cover the demand for diagnostic imaging services [4].

In our previous paper, “Mentoring in radiology: an asset worth exploring,” we provided guidance on enhancing career development, acquiring skills and achieving professional satisfaction, as well as insights into faculty retention [5]. Mentoring in radiology encompasses various strategies and models, including formal and informal approaches, as well as individual, group and peer mentoring. Integrating these diverse models can result in a comprehensive approach known as ‘mosaic mentoring’ [6].

We now propose the Cascade Mentoring model (senior → junior → student), which has been used in other healthcare fields [7]. However, to our knowledge, it has never been formally implemented in radiology education before.

In line with the European training curriculum, this proposal supports the implementation of a Cascade Mentoring model in radiology training to enhance peer and near-peer learning, develop teaching skills and increase undergraduate engagement in radiological sciences [8]. As has been wisely observed: “Who better to get advice from than a senior resident or junior faculty member who has already been through the process and understands the challenges of being a radiology resident?”

## Objectives of cascade mentoring

The main objectives of cascade mentoring are as follows:

1. Align teaching and mentorship with the European training curriculum, ensuring progression from Level I to Level III.
2. Empower residents with teaching, leadership and mentoring skills.
3. Provide undergraduates with structured early exposure to radiology, fostering interest and foundational knowledge.
4. Optimise faculty time and resources by distributing teaching and mentorship responsibilities.
5. Promote a culture of sustainable, continuous mentorship, peer learning and teaching excellence.
6. Enhance training capacity and support faculty retention.

## Concept model and structure of cascade mentorship

We propose a cascade mentorship model based on the pyramid concept, where the roles of mentor and mentee merge. We believe this model is the most effective way for senior advisors to pass on their knowledge to residents and undergraduates.

**Fig. 1:**
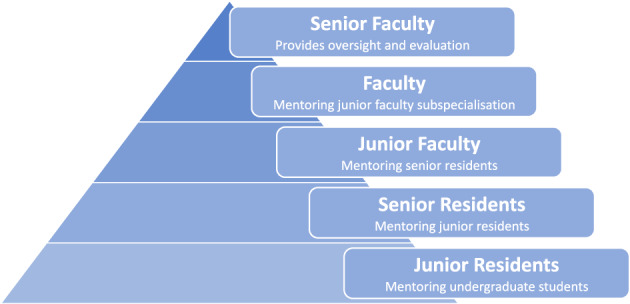
Cascade mentoring pyramid

Flowchart of cascade mentorship and duties (Fig. 1):

– Senior faculty radiologists (department and subspecialty division heads): ensure alignment with the European training curriculum (ETC), provide oversight and evaluate the mentoring programme.

- Faculty radiologists (Subspecialists): Responsible for subspecialty mentorship (ETC level III) for early career radiologists (junior radiologists).
- Junior faculty (first 3–5 years after receiving specialist title): Mentor senior residents.
- Senior residents (years 4–5): Mentor junior residents and support them in advanced reporting, clinical judgement and research.
- Junior residents (years 1–3): Mentor undergraduate medical students in the basics of image interpretation and case discussions.
- Undergraduate students: Engage in case-based learning and anatomy/imaging correlation.

This layered approach promotes peer-assisted learning and leadership development, as well as the vertical integration of teaching across training levels. It effectively redistributes the educational workload across the trainee hierarchy in the form of a cascade mentoring pyramid.

## Cascade mentoring’s integration with the European training curriculum (ETC)

The European training curriculum defines three teaching levels:

- Level I (Years 1–3): Teaching of the foundations of general radiology across all domains.
- Level II (Years 4–5): Advanced clinical training with elective rotations.
- Level III: Post-graduation level. Specialisation in one of the following areas: breast, cardiac, thoracic, musculoskeletal, gastrointestinal, gynaecological, head and neck, paediatric, urogenital, neuroradiology, interventional, emergency, oncological or imaging informatics.

The ETC covers areas such as knowledge, skills, attitudes, professionalism, research and communication. Integrating cascade mentoring across these areas is mutually beneficial, fostering a win–win dynamic for all participants.

The benefits of teaching junior trainees enable senior residents to consolidate their mastery, while guiding undergraduates reinforces foundational knowledge for junior residents. Competencies such as image interpretation, reporting, procedural techniques and diagnostic reasoning are developed progressively: undergraduates acquire core fundamentals, junior residents refine their skills, and senior residents achieve advanced proficiency while assuming teaching responsibilities. Mentors also guide mentees through research projects, evidence-based practice and exam preparation, including the European Diploma in Radiology (EDiR), which is aligned with the European training curriculum [9].

## Proposal for the implementation model of cascade mentoring in radiology aligned with ETC

We propose a structured implementation model using a phased approach from planning to execution and evaluation. In the event of a positive evaluation, the cascade mentoring pyramid can be disseminated. The subsequent section will provide illustrative examples of best practices as outlined in the literature.

### Phase 1: Planning and governance

The establishment of a Mentorship Steering Committee is imperative, comprising senior faculty members (department chairs and division heads), mid-level and junior faculty members and resident representatives. The committee bears responsibility for the allocation of the requisite resources to ensure alignment with the European training curriculum (ETC). The document also establishes a framework of policies, delineates the respective roles of the parties involved and establishes a set of Key Performance Indicators (KPIs) to assess the activities undertaken within the programme. This provides the committee with a comprehensive evaluation of both the quantity and quality of the programme’s output. This information can then be used to inform strategic decisions and achieve the objectives of the mentorship programme [10].

The Committee will define the roles, responsibilities and objectives of the programme. For each tier of the academic hierarchy (senior faculty, junior faculty, senior residents, junior residents and students), the committee will establish specific, measurable, achievable, relevant and time-bound (SMART) objectives [11].

A kick-off workshop should be organised to introduce the programme, its roles and responsibilities and the associated expectations and timeline (SMART objectives) (Table 1).

**Table 1:** Examples of SMART objectives by tier

	Examples of SMART objectives
Junior faculty	Conducting at least six formal mentoring meetings with the assigned residents each academic year.
Senior residents	Leading twelve case-based teaching sessions for junior residents per year, with peer evaluation.
Junior residents	Running ten image-interpretation workshops for students per year, and using pre- and post-session quizzes to assess learning.

The programme encompasses mentor training and the development of teaching capacity. Before the initiation of the programme, structured workshops on mentorship pedagogy, feedback, role modelling, diversity, inclusion and equity should be provided for all mentors (residents, junior faculty and senior faculty). A recent scoping review found that effective mentor training programmes improve mentor quality and mentee outcomes, thus emphasising the critical role of mentor training [12]. Mentors at each tier undergo training, while mentees receive structured guidance, with teaching and leadership skills being cultivated concurrently.

The development of formal mentorship materials is recommended. The creation of mentorship handbooks or guides is recommended, outlining the expectations for each tier. These should include meeting templates, feedback forms and developmental milestones (aligned with ETC). A structured checklist should be provided for each mentor–mentee interaction. The checklist should include items such as the initial meeting, the mid-year review and the end-of-year reflection.

The provision of institutional support and the existence of incentives are of significance in this context. In order to consolidate the programme, it is recommended that recognition of mentorship duties be considered in faculty evaluations and promotion criteria, as well as through the introduction of a formal ‘mentorship’ award/certificate. The allocation of dedicated time for mentorship activities (e.g., 1–2 h per week) within faculty and resident schedules is also recommended to overcome the frequently cited time barrier “no time for mentoring” and to sustain engagement through formal recognition [13, 14].

### Phase 2: Pilot and execution

We propose an execution pilot phase of 1–12 months. The initiation of the programme should commence with a pilot cohort, for example, one division, in order to trial the cascade model. To illustrate this point, the Musculoskeletal (MSK) division is worthy of consideration. The mentoring scheme at MSK is structured such that a senior faculty specialist mentors junior faculty fellows (level III ETC), junior faculty mentors senior residents (level II ETC), senior residents mentor junior residents (level I ETC), and junior residents mentor medical students in the U-level curriculum.

The implementation of near-peer mentoring (senior residents → junior residents → residents → students) has garnered significant support within the domain of medical education [14]. This approach has been demonstrated to develop research skills and mentorship capacity while distributing the workload [15].

**Fig. 2:**
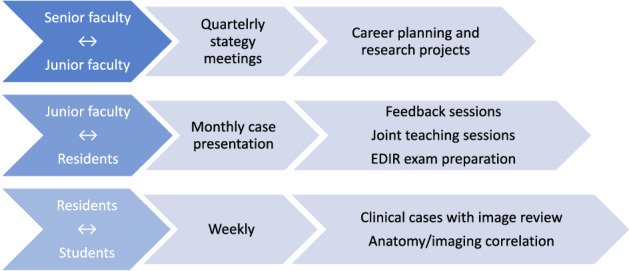
Structured meetings and activities

Structured meetings and activities with regular interactions should be scheduled for each mentorship layer, as shown in Fig. 2.

A true Community of Practice (CoP) model should be established, based on regular group meetings that encourage peer learning and foster a sense of shared identity. There is evidence to suggest that peer mentorship promotes a sense of belonging, professional identity and mutual engagement [16]. This sense of community can be reinforced through informal meet-ups, such as ‘Mentor Café’ sessions or reflection forums, where mentors at different levels can meet to discuss challenges and share experiences. This CoP model fosters a lasting mentoring culture, which is crucial for ensuring the programme’s continuity in the future.

### Phase 3: Evaluation and feedback

Programme evaluation is of paramount importance. At the end of the first year, it is time to analyse the programme, using metrics and surveys to drive iterative improvement, ensuring the programme evolves and remains effective.

Examples of quantitative metrics that can be used to measure programme effectiveness include the number of mentor–mentee meetings and teaching sessions, the number of research projects started or completed, research outputs (publications) and EDiR pass rates [17].

Qualitative metrics, such as satisfaction surveys, focus groups and open feedback sessions, are also important [18].

The satisfaction of mentors and mentees should also be evaluated using validated instruments. For example, survey tools used in previous radiology mentoring studies, such as those published by Bredella et al or Kashiwagi et al, could be adapted [12, 19].

The programme should be dynamic and refined as required. Periodic peer reviews of teaching and mentorship sessions should also be conducted to ensure continuous quality improvement. Open feedback should be encouraged. If mentees report a lack of structure in the presentations, adjust the meeting templates accordingly. Likewise, if mentors report time constraints, the scheduling should be revised [20].

### Phase 4: Scaling-up and sustainability

After a successful 1-year programme in one subspecialty, it’s time to scale up to additional subspecialties and/or sites. The programme can be expanded to include other divisions, such as neuroradiology or abdominal imaging, in line with ETC Level III. Another option is extending the programme to affiliated hospitals. At this time, more junior faculty and senior residents should be recruited, and mid-career faculty members should be encouraged to act as mentors in their respective subspecialties, thereby strengthening subspecialty mentorship capacity.

Sustaining motivation is a cornerstone for the success and future of this teaching model. One easy and inexpensive way to maintain motivation is through recognition. Other effective strategies include formalising mentorship roles, such as ‘Mentorship Advisor, allocating protected time and providing academic recognition in the form of certificates or awards.

Adopting the Cascade Mentoring policy and strategy can continually fuel a Mentoring-of-Mentors pipeline, in which resident mentors train the next generation in a sustainable and self-reinforcing way.

The Table 2 is a summary of the implementation logic of the Cascade Mentoring model.

**Table 2:** Logic of the cascade mentoring model

1. Alignment with the European training curriculum (ETC): By having mentors and mentees distributed across levels, the cascade mentoring model supports progression through the ETC levels (I, II and III).
2. Build capacity: Residents, not just faculty, play an active role in mentorship, particularly in teaching and research, thereby distributing the workload.
3. Developing skills: Mentors at each level receive training and mentees receive structured guidance, cultivating teaching and leadership skills.
4. Sustainability: The Cascade Mentoring Community of Practice and its recognition build a lasting mentoring culture, ensuring continuity.

## Challenges and proposed solutions in cascade mentoring

Cascade mentoring in radiology can enhance the clinical skills and teaching competencies of residents and faculty members alike. Studies have shown that mentoring by residents can improve the outcomes for both mentors and mentees [21]. However, its implementation is often hindered by operational and structural challenges. Table 3 summarises the main challenges and potential solutions derived from the authors’ departmental experience and the literature.

**Table 3:** Key challenges and proposed solutions in cascade mentoring

Challenge	Proposed solution
Time constraints for residents/faculty	Schedule mentoring sessions within working hours and limit each session to 20–30 min [12]
Ensuring the quality and consistency of cascade mentoring	Provide mentor training workshops Organise mentoring guides and faculty oversight Implement peer review of teaching sessions [6, 22]
Lack of residents‘ teaching experience	Offer teaching skills workshops for residents Run a mentorship-of-mentors program to prepare residents as junior mentors [6, 23]
Sustaining motivation	Recognise mentors: certificates, academic credits Integrate mentoring into departmental culture

By addressing these challenges systematically, cascade mentoring programs can maintain high-quality interactions and measurable outcomes, facilitating accountability and continuous feedback.

Cascade Mentoring can also play a positive role in mitigating the challenge of a growing shortage of radiologists in Europe, which is affecting healthcare quality and training [22]. EU-REST (European Union Radiation, Education, Staffing and Training) data reveal substantial disparities in radiologist density, ranging from 51 per million in Bulgaria to 270 in Sweden, with 16 of 27 countries below the EU average of 127 [23, 24]. Another issue is the ageing workforce in Europe (around 45% over 51 years), threatening to worsen shortages compounded by increasing subspecialisation, expanding imaging modalities and limited faculty availability [3]. Cascade mentoring offers a scalable solution by enhancing training capacity and supporting retention of early-career radiologists.

To provide a comprehensive diagnostic of the proposed model, Table 4 summarises the internal and external factors via a SWOT (strengths, weaknesses, opportunities, threats) analysis, highlighting the strategic advantages and potential implementation challenges of Cascade Mentoring in the current radiological landscape.

**Table 4:** SWOT analysis of cascade mentoring proposal

Strengths	Weaknesses
• **Curricular alignment**: Directly integrated with the European training curriculum (ETC).	• **Time constraints:** Heavy clinical workloads for both faculty and residents.
• **Scalability**: Sustainable framework distributing teaching across all levels.	• **Inexperience:** Junior mentors may lack formal pedagogical training.
• **Mutual benefit**: Knowledge consolidation for seniors; structured guidance for juniors.	• **Operational complexity:** Requires a Governance Committee and rigorous oversight.
• **Professional development**: Early development of leadership and teaching skills.	

Opportunities	Threats
• **Workforce retention**: Increases professional satisfaction to mitigate staff shortages.	• **Cultural resistance:** Traditional hierarchies may resist the new mentoring model.
• **Recruitment**: Early undergraduate engagement attracts students to the specialty.	• **Motivation sustainability:** Risk of losing interest without formal recognition or awards.
• **Digital integration**: Use of SMART objectives and digital feedback tools.	• **Implementation variability:** Inconsistent outcomes due to resource disparities across countries.
• **Community building**: Fosters a sense of belonging through a Community of Practice	

## Bridging the gap: the role of EDiR and cascade mentoring in European radiology harmonisation

In an increasingly multicultural Europe characterised by diverse healthcare landscapes, the advancement of radiological excellence relies on the synergy between formal certification, international exposure and the contribution of a robust cascade mentoring model. At the foundational level, the European Diploma in Radiology (EDiR) serves as a crucial instrument for the harmonisation of radiological education. By aligning its standards with the European training curriculum (ETC), the EDiR provides a unified objective benchmark that ensures a consistent level of proficiency across national training programs, establishing the necessary baseline for effective knowledge transfer [1, 25].

Building upon this core qualification, recognised subspecialty diplomas, such as the European Diploma in Musculoskeletal Radiology (EDiMSK) and the European Diploma in Breast Imaging (EDBI), and others, are instrumental in validating specialised expertise. These certifications provide the advanced benchmarks required for senior mentors to guide their peers [25, 26].

In addition, international scholarships—such as those from the European School of Radiology (ESOR)—and specialised summer schools (e.g., ESGAR, ESUR) serve as catalysts for growth. These programs allow mentors to navigate different clinical systems, importing international best practices back into their local mentoring chain.

This cascade mentoring model is particularly transformative for junior residents. In this structured hierarchy, senior specialists who have achieved international certification pass their refined diagnostic skills and best-practice mindsets down to senior residents, who in turn mentor junior trainees [27, 28].

By integrating rigorous standardised certification with immersive global training, the Cascade model may help mitigate cultural and systemic disparities, fostering a resilient, unified professional community and a continuous cycle of improvement across the European radiological landscape [29].

## Conclusion

Cascade mentoring provides an innovative, scalable model that strengthens radiology education in Europe. Aligned with the ESR curricula at all levels, it fosters teaching and leadership skills among residents and junior radiologists, thereby benefiting the department as a whole. Implemented, overseen and evaluated thoughtfully, this model has the potential to enhance the quality of radiology education, institutional capacity and professional development in both clinical and academic settings. Cascade mentoring can also help mitigate the issue of the shortage of radiologists by enhancing training capacity and retention.

## Abbreviations

CoP: Community of Practice
EDiR: European Diploma in Radiology
ESR: European Society of Radiology
ETC: European training curriculum for radiology
EU: European Union
MSK: Musculoskeletal
SMART: Specific, measurable, achievable, relevant and time-bound
SWOT: Strengths, weaknesses, opportunities, threats
U-level: Undergraduate level

## References

[1] European Society of Radiology (2025) European training curriculum for radiology—curriculum for the Level I and II training programme. Available via https://www.myesr.org/app/uploads/2025/02/ESR_2025_European_Training_Curriculum_Level_I_II.pdf. Access date: September 2025

[2] European Society of Radiology (2024) European training curriculum for subspecialisation in radiology. Available via https://www.myesr.org/app/uploads/2024/02/ESR_2024_European_Training_Curriculum_Level_III.pdf. Access date: September 2025

[3] European Society of Radiology (2022) Attracting the next generation of radiologists: a statement by the European Society of Radiology (ESR). Insights Imaging 13:84 10.1186/s13244-022-01221-8 PMC9066129 35507198

[4] The Royal College of Radiologists Clinical Radiology (2025) Clinical radiology workforce census 2024. Available via https://www.rcr.ac.uk/media/5befglss/rcr-census-clinical-radiology-workforce-census-2023.pdf. Access date: November 2025

[5] Vieira A, Cabri MM, Spijkers S, Vieira AC, Maas M (2022) Mentoring in radiology: an asset worth exploring! Eur J Radiol 155:110133 34991912 10.1016/j.ejrad.2021.110133

[6] Khatchikian AD, Chahal BS, Kielar A (2021) Mosaic mentoring: finding the right mentor for the issue at hand. Abdom Radiol (NY) 46:5480–5484 34716779 10.1007/s00261-021-03314-2 PMC8556786

[7] Ajeani J, Mangwi Ayiasi R, Tetui M et al (2017) A cascade model of mentorship for frontline health workers in rural health facilities in Eastern Uganda: processes, achievements and lessons. Glob Health Action 10:1345497 28816629 10.1080/16549716.2017.1345497 PMC5645691

[8] Frei E, Stamm M, Buddeberg-Fischer B (2010) Mentoring programs for medical students—a review of the PubMed literature 2000–2008. BMC Med Educ 10:32 20433727 10.1186/1472-6920-10-32 PMC2881011

[9] European Board of Radiology (2025) European Diploma in Radiology (EDiR): all about the examination. Available via https://www.myebr.org/edir/all-about-the-examination

[10] Bento RF, Gandara MER, Penteado SP (2025) Key performance indicators applied in medicine. Int Arch Otorhinolaryngol 29:1–10 41019826 10.1055/s-0045-1809426 PMC12473520

[11] Chatterjee D, Corral J (2017) How to write well-defined learning objectives. J Educ Perioper Med 19:E610 29766034 PMC5944406

[12] Bredella MA, Alvarez C, O’Shaughnessy SA, Lavigne SD, Brink JA, Thrall JH (2021) Radiology mentoring program for early career faculty—implementation and outcomes. J Am Coll Radiol 18:451–456 33031784 10.1016/j.jacr.2020.09.025 PMC7935755

[13] Grayev A (2025) Near peer mentoring as an opportunity for trainees and departments to thrive. J Am Coll Radiol 22:1303–1307 40639587 10.1016/j.jacr.2025.07.001

[14] Oak S, Glickman C, McMackin K (2025) Near-peer mentorship: promoting medical student research with resident pairing. J Med Educ Curric Dev 12:23821205251329659 40103580 10.1177/23821205251329659 PMC11915252

[15] Lakhani DA, Swaney KJ, Hogg JP (2022) “Resident managed peer-mentoring program”: a novel way to engage medical students and radiology residents in collaborative research. Acad Radiol 29:1425–1431 34863631 10.1016/j.acra.2021.11.004 PMC9156656

[16] Lim JJ, Verbo VA, Khandelwal G, Nograles NH (2025) Peer mentoring as a community of practice in medical education. Clin Teach 22:e70238 41192820 10.1111/tct.70238 PMC12588802

[17] Monday LM (2022) Define, measure, analyze, improve, control (DMAIC) methodology as a roadmap in quality improvement. Glob J Qual Saf Healthc 5:44–46 37260837 10.36401/JQSH-22-X2 PMC10229001

[18] Guo T, Chowdhury M, Rasouli R, Patel M (2023) Exploring the effectiveness of a cascading mentorship model in developing CanMEDS competencies in postgraduate medical education: a qualitative interview study among resident mentors at a medical school in Canada. BMJ Open 13:e061338 36631235 10.1136/bmjopen-2022-061338 PMC9835859

[19] Kashiwagi DT, Varkey P, Cook DA (2013) Mentoring programs for physicians in academic medicine: a systematic review. Acad Med 88:1029–1037 23702518 10.1097/ACM.0b013e318294f368

[20] Horowitz JM, Choe MJ, Dienes K et al (2022) Team approach to improving radiologist wellness: a case-based methodology. Curr Probl Diagn Radiol 51:806–812 35365374 10.1067/j.cpradiol.2022.02.006 PMC9356970

[21] Kikano EG, Ramaiya NH (2022) Mentorship in academic radiology: a review from a trainee’s perspective. *Radiology* in training. Radiology 303:E17–E19 35103538 10.1148/radiol.212205

[22] Brady AP, Loewe C, Brkljacic B et al (2025) Guidelines and recommendations for radiologist staffing, education and training. Insights Imaging 16:57 40074928 10.1186/s13244-025-01926-6 PMC11904080

[23] European Health and Digital Executive Agency (European Commission) (2025) Analysis on workforce availability, education and training needs for the quality and safety of medical applications involving ionising radiation in the EU. Status and recommendations—final report. Publications Office of the European Union

[24] Brady AP, Paulo G, Brkljacic B et al (2025) Current status of radiologist staffing, education and training in the 27 EU member states. Insights Imaging 16:59 40088348 10.1186/s13244-025-01925-7 PMC11910488

[25] European Board of Radiology (2018) The European Diploma in Radiology (EDiR): investing in the future of the new generations of radiologists. Insights Imaging 9:905–909 10.1007/s13244-018-0665-7 PMC6269329 30291525

[26] Snoj Z, Hebar T, Sconfienza LM et al (2020) Present status of musculoskeletal radiology in Europe: international survey by the European Society of Musculoskeletal Radiology. Semin Musculoskelet Radiol 24:323–330 32987429 10.1055/s-0040-1713119

[27] Gregory J, Kofoed-Ottesen M, Lindlbauer B, Loewe C, Vilgrain V, European Society of Radiology (2025) Assessing the perceived impact of ESOR training programs on radiologists’ professional development. Insights Imaging 16:34 39961923 10.1186/s13244-024-01891-6 PMC11833024

[28] Hadian F, Din F, Butt N, Stott S, Navarro OM (2024) International postgraduate fellowships in radiology: the good, the bad, and the paperwork. BJR Open 6:tzad010

[29] Adriaensen M, Ricci P, Loewe C et al (2025) Subspecialisation recognition in European radiology—follow-up survey by the Accreditation Council in Imaging and European Society of Radiology National Societies Committee. Insights Imaging 16:180 40815381 10.1186/s13244-025-02054-x PMC12356789

